# High Frequency Variations of Earth Rotation Parameters from GPS and GLONASS Observations

**DOI:** 10.3390/s150202944

**Published:** 2015-01-28

**Authors:** Erhu Wei, Shuanggen Jin, Lihua Wan, Wenjie Liu, Yali Yang, Zhenghong Hu

**Affiliations:** 1 School of Geodesy and Geomatics, Wuhan University, Wuhan 430079, China; E-Mails: lh.wan@whu.edu.cn (L.W.); rjsq@whu.edu.cn (W.L.); yangyl@whu.edu.cn (Y.Y.); hzh597@whu.edu.cn (Z.H.); 2 Department of Geosmatics Engineering, Bulent Ecevit University, Zonguldak 67100, Turkey; 3 Shanghai Astronomical Observatory, Chinese Academy of Sciences, Shanghai 200030, China; 4 Earth Oriented Space Science and Technology, Technische Universität München, Munich 80333, Germany

**Keywords:** Earth Rotation Parameters, high frequency variation, ocean tide

## Abstract

The Earth's rotation undergoes changes with the influence of geophysical factors, such as Earth's surface fluid mass redistribution of the atmosphere, ocean and hydrology. However, variations of Earth Rotation Parameters (ERP) are still not well understood, particularly the short-period variations (e.g., diurnal and semi-diurnal variations) and their causes. In this paper, the hourly time series of Earth Rotation Parameters are estimated using Global Positioning System (GPS), Global Navigation Satellite System (GLONASS), and combining GPS and GLONASS data collected from nearly 80 sites from 1 November 2012 to 10 April 2014. These new observations with combining different satellite systems can help to decorrelate orbit biases and ERP, which improve estimation of ERP. The high frequency variations of ERP are analyzed using a de-trending method. The maximum of total diurnal and semidiurnal variations are within one milli-arcseconds (mas) in Polar Motion (PM) and 0.5 milli-seconds (ms) in UT1-UTC. The semidiurnal and diurnal variations are mainly related to the ocean tides. Furthermore, the impacts of satellite orbit and time interval used to determinate ERP on the amplitudes of tidal terms are analyzed. We obtain some small terms that are not described in the ocean tide model of the IERS Conventions 2010, which may be caused by the strategies and models we used or the signal noises as well as artifacts. In addition, there are also small differences on the amplitudes between our results and IERS convention. This might be a result of other geophysical excitations, such as the high-frequency variations in atmospheric angular momentum (AAM) and hydrological angular momentum (HAM), which needs more detailed analysis with more geophysical data in the future.

## 1. Introduction

The Earth Rotation Parameters (ERPs) are changing due to geophysical excitation (such as redistribution of geophysical fluid mass) as well as lunisolar gravitational torque, including the motion of the rotation axis (Polar motion, PM) and its change rate (Length of Day, LOD). In addition, some larger crustal activities may also affect Earth's rotation. Hopkin [1] and Wu *et al.* [2] found that the Sumatra earthquake and Pleistocene deglaciation made the Earth's rotation rate change by several microseconds, respectively. The detailed theoretical mechanism accounting for the variations in Earth rotation was a hot issue over the past decades from decades to daily variations in Earth Rotation [3]. At present, several different approaches have been suggested for predicting and estimating high frequency variations. In previous research, variations in Earth Rotation from oceanic tides were predicted based on theoretical tidal [4] and hydrodynamical models [5]. The effects from geophysical excitation over long periods rather than days were studied at first. Then, shorter periods such as P1, K1 and O1 in the diurnal band and M2, S2 and N2 in semidiurnal band were considered [6–9]. In 1991, the stable Very Long Baseline Interferometry (VLBI) was first used to estimate variations and the effect of tides on Universal Time (UT) [10]. The coefficients of tidal amplitude for PM and UT then were estimated from VLBI data [11–17] and combined VLBI and GPS observations [18] as well as combined VLBI and ring laser observations [19]. Satellite Laser Ranging (SLR) data were also used to estimate the variations in ERPs [20]. At the same time, Ray *et al.* [21] and Chao *et al.* [22] predicted variations in ERP from a new tide model induced from TOPEX/Poseidon altimeter data. More recently, GPS data were applied to high frequency variations for ERP. The agreement between different techniques was at a level of 10–30 μas in PM and 1–3 ms in UT1 [23], followed by Steigenberger *et al.* [24]. Then, the effects of ocean and hydrology in PM and LOD also have been investigated from the observations of GRACE Satellites by Jin *et al.* [25–27], followed by Panafidina [28] and then the interactions between GPS orbits and ERP were investigated.

The International Earth Rotation and Reference System Service (IERS) estimates ERPs at a sub-millimeter precision. Using VLBI, SLR, Lunar Laser Ranging (LLR), GPS, and Doppler Orbitography by Radiopositioning Integrated on Satellite (DORIS). However, Most of the IERS ERP series, such as IERS C04 [29] and Bulletin A (rapid prediction ERP series) do not contain high frequency variations because they are smoothed by Vondrak filtering [30,31]. Recently, with improvement of GPS and GLONASS observation precision and networks, it provides a new opportunity to estimate high frequency variations of ERP. In this paper, ERP series with a time resolution of one hour are computed from GPS, GLONASS, and combined GPS and GLONASS observations. While combining both GPS and GLONASS will decrease the correlation between UT1 and orbit model since GPS and GLONASS has different orbital period. Then a de-trending method based on a smoothness priors approach is employed to obtain high frequency variations in ERP. Furthermore, these high frequency variations are analyzed with Fourier transform to investigate the components of tidal terms hidden in the variations. In addition, the impact of time interval used to estimate ERP, different strategies and models on tidal amplitudes are also discussed.

## 2. Data and Processing

### 2.1. GPS/GLONASS Observations

Up to now, about one hundred stations are available to track both GPS and GLONASS satellites simultaneously. The precision of ERP is related more to the distribution of the satellites than the number of stations [32] and does not improve much when the number of sites increases over 60 according to Wei *et al.* [33]. Here, about 80 sites from International GNSS Service (IGS) [34] are selected to estimate ERP.

**Figure 1.**
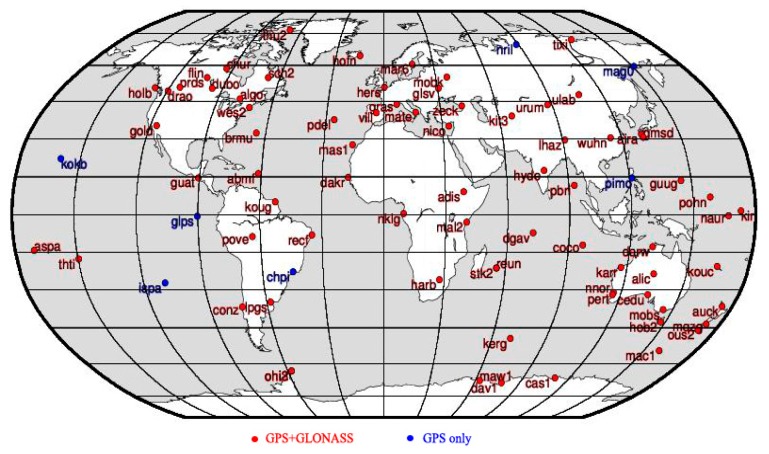
Distribution of GPS and GLONASS stations in this study. Red are the stations that track both GPS and GLONASS system. Blue are the station only track GPS system.

First of all, these stations are core sites of International Terrestrial Reference Frame (ITRF) [35]. Second, the standard deviation of coordinates of the sites is less than 1 mm, while the standard deviation of velocity of the sites is less than 0.2 mm per year according to the publications of IGS. Finally all the stations satisfy a uniform distribution with stable and high quality observations. The distribution of these stations is shown in Figure 1 and observations of 526 days from these stations are collected since 11 January 2012.

### 2.2. Data Processing Models

The uninterrupted and continuous tracking stations established by IGS allow us to accumulate a large number of observations from GPS and GLONASS to estimate ERP from a few hours of data. This is a great advantage when compared to other technologies, such as SLR or VLBI, which does not have continuous observations. In this paper, ERPs are estimated for every hour with GPS and GLONASS observations, individually and by a combined GPS and GLONASS method. The theory and methods to determinate ERP were introduced in details in the references [32,36,37].

All GNSS data analysis was executed using Bernese 5.0 software [38] with double-difference observation. Apart from ERPs, the coordinates for sites, the troposphere zenith delays, initial phase ambiguities, and station clock errors were also taken into account when processing. The orbit was strongly constrained to the IGS precision ephemeris and some other models were used: The IERS2000 sub-daily PM model together with the IAU2000 Nutation model [39], the OT_CSRC ocean tide file [40] and the FES2004 Ocean loading correction [41]. Because troposphere delays differ from time to time, especially for some rapidly changing tropospheric conditions, if the tropospheric delay is not estimated at a sufficient temporal resolution, then parts of the delays will propagate into the ERP. At the same time, the sampling interval for the troposphere delays may have influences on diurnal and semidiurnal tidal periods. As a result, extreme care must be taken when sampling the troposphere delays. In this paper, site-specific troposphere delays were estimated every hour using the WET NIELL [42] mapping function. Additionally, the quasi-ionosphere-free (QIF) model [43] was deployed to deal with initial phase ambiguities.

In the estimation of ERP, we divide a long interval (e.g., one day or 3 day arc) into several sub-interval of equal length (2 or 4 h). Then in any sub-interval [*t_i_*,*t_i_*_+1_], the ERP can be represented as following:

(1) $$\text{ERP}(t)={\text{ERP}}_{i}+E\dot{R}P_{i}(t-t_{i})$$

where *ERP_i_* and *EṘP_i_* is the offset and drift in the sub-interval, respectively. The first offset of UT1-UTC of each long interval has been constrained to a prior value (Bulletin A) since the correlation between UT1-UTC and orbital parameters. For UT1-UTC, the drift *EṘP_i_* actually is the rate and can be represented by –LOD. Furthermore, the constraint of continuity at the sub-interval boundary is added according to Equation (2):

(2) $${\text{ERP}}_{i+3}-({\text{ERP}}_{i}+E\dot{R}P_{i}(t_{i+3}-t_{i}))=0$$

**Figure 2.**
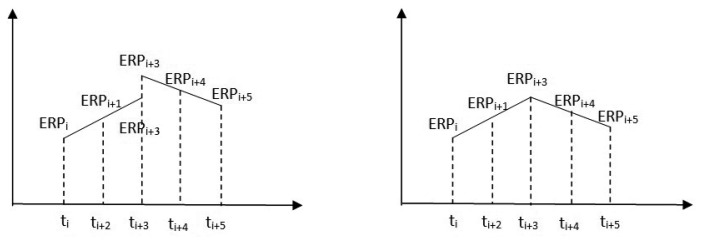
The principle of estimating hourly ERPs before (**left**) and after (**right**) the constraint added. Notice that the ERP_i+3_ in the left are not equal to the ERP_i+3_ in the right.

Figure 2 shows the principle of estimating ERPs and the constraint added to the interval borders. According to these conditions, we actually obtained an ERP solution every hour when the length of sub-interval is 2 h, but only 13 of them are independent. However, we will only obtain 12 sets of ERP with seven of them are independent when the length of sub-interval is 4 h, that is to say, the temporal resolution of ERP becomes two hour. By the way, because the sub-daily resolution of ERP will lead to singularity due to the correlations between daily retrograde motion of pole and the orientation of satellites orbital planes, as a consequence, the retrograde diurnal component in PM has to been blocked if ERPs are determined together with orbital elements. This can be completed by blocking it or removing it with a numerical filter. We refer readers to [3] for more information. In order to decrease the impacts of satellite orbit on diurnal terms of ERP, we also operate a computation of ERPs with long arc by combining the normal equations every three days.

## 3. Variations in ERP

### 3.1. ERP Results from GPS and GLONASS

According to the strategy described in Section 2.2, firstly, we estimate the daily ERP for one month and then compare them to IGS published values in order to assess the accuracy of our process strategy. The Root Mean Square (RMS) difference of PM and UT1-UTC is about 0.23 mas (0.31 mas) and 0.017 ms (0.027 ms) when compared our daily ERP from GPS (GLONASS) to the IGS values, respectively. These figures show a result with a good enough precision when considering that only about 80 sites are involved. Then ERPs are estimated with a frequency of 1 h for 526 days since 11 January 2012, but only the independent sets of ERPs are used here, that is to say the ERP are used every 2 h or 4 h. To give us a first indication of how larger is the high frequency variations, the ERP series are compared to IGS published values, too. This seems to be a strange comparison, because the ERP estimated with a sub-interval of 2 h from GPS, GLONASS and combined GPS + GLONASS contains many subdaily signals, however, the IGS published values do not contain the subdaily signal. Thus, to operate an appropriate and informative comparison, we firstly compute the daily average of the hourly ERPs for each day. Then the daily average series of ERPs are compared with the IGS values (at UTC 12:00:00). As we note, this comparison was operated only to give us a first indication of how larger is the high frequency variations. The statistic information of the differences between daily average results and IGS published values (at UTC 12:00:00) are shown in Table 1. It is clear that the precision of ERP from GPS is much higher than that from GLONASS. The result of ERP was improved distinctly by combined GPS and GLONASS. Furthermore, to our best knowledge, bringing in GLONASS observations can also reduce the impact of orbit model on ERPs. Because the period of GPS orbits is 12 h, which is identical to the semidiurnal tidal terms. Therefore, estimating ERP with GLONASS (period of GLONASS satellites is 11 h and 15 min) may help to reduce the correlation between orbit model and ERP which will be discussed at Section 3.3. The accuracy of PM in X and Y from combined GPS and GLONASS were improved by about 6%∼7% when compared to GPS only. Dach *et al.* [44] shows a similar improvement when estimate the positions. The accuracy of UT1-UTC is not as good as PM and combined result is slightly off. The most possible explanation for the accuracy of UT-UTC is that, as we mentioned before, we cannot estimate the UT1-UTC in an absolute sense. Thus, we introduce additional information (the first UT1-UTC offset (at UTC 00:00:00) of each long interval has been constrained to a prior value) so as to estimate the UT1-UTC. This method then leads to a little systematic error and a less improvement because only the UT1 rate is accessible to GPS/GLONASS, as a result, we actually need an offset value for each sub-interval if high precise UT1-UTC is expected.

### 3.2. High Frequency Variations in ERP Series

The ERP series directly estimated from GPS and GLONASS contains both low and high frequency variations. Apart from the subdaily variations, a long period term is also included in the ERP series we acquired. To obtain the subdaily variations in ERPs, the long period term is removed in the ERPs generated from GPS and GLONASS. This can be accomplished by subtracting a prior trend which is also an ERP series but without high frequency variations [23]. IERS C04 and Bulletin A published by IERS is the optional prior ERP series which do not contain high frequency variations after handling with Vondrak filtering. However, IERS C04 offers only one ERP per day. So, first of all, if IERS C04 is used as a prior ERP, we need to densify the C04 series to one ERP solution per hour through Lagrange interpolation because the time resolution of the ERP series we estimated is one hour. However, this Lagrange interpolation may add interpolation error. In this paper, we use a de-trending method based on a smoothness priors approach operating like a time-varying (Finite Impulse Response) FIR high pass filter [45]. Firstly, the entire ERP series can be considered to consist of two components:

(3) $$S_{\text{ERP}}=S_{\text{high}\_\text{freq}}+S_{\text{trend}}$$

where *S_high_freq_* is the high frequency variations of ERP and *S_trend_* is the trend of ERP which can be modeled as:

(4) $$S_{\text{trend}}=H\theta +\upsilon$$

where *H* is the observation matrix, *θ* are the regression parameters and *υ* is the observation error. Then the main task is to estimate the parameters *θ* by some fitting procedure. The most widely used method is the Least Square Method. Here, [45] provides a more general approach called regularized least square as follows:

(5) $${\hat{\theta }}_{\lambda }=\arg\underset{\theta }{\min}\{{\left\|H\theta -z\right\|}^{2}+\lambda^{2}{\left\|D_{d}(H\theta )\right\|}^{2}\}$$

where *λ* is the regularization parameter and *D_d_* indicates the discrete approximation of d'th derivative operator. The observation matrix *H* can be denoted according to some known properties of ERP series, but here *H* is denoted as identity matrix. Finally, the solution can be rewritten as:

(6) $${\hat{\theta }}_{\lambda }={\left(H^{T}H+\lambda^{2}H^{T}D_{d}^{T}D_{d}H\right)}^{-1}H^{T}S_{\text{ERP}}$$

(7) $$S_{\text{trend}}=H{\hat{\theta }}_{\lambda }$$

**Figure 3.**
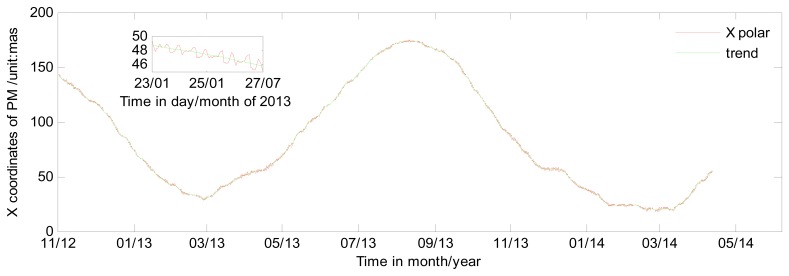
Long term variation of PM in X coordinate form GPS with 2 h sub-interval. Upper left is the enlargement section of 23–27 January 2013. Red line represents the original ERP series and green line represents the trend which is considered as long term variation.

Figure 3 shows the detail effects of the de-trending method. Upper left is the enlarged sub-figure of the ERP series from 23–27 January 2013. From Figure 3, we can see the de-trending method of smoothness priors approach performs very well in fitting the trends of ERP series, and the trends we acquire are very similar to IERS C04. Then the high frequency variations of ERP are generated by subtracting the trend from the original ERP series we estimate.

**Figure 4.**
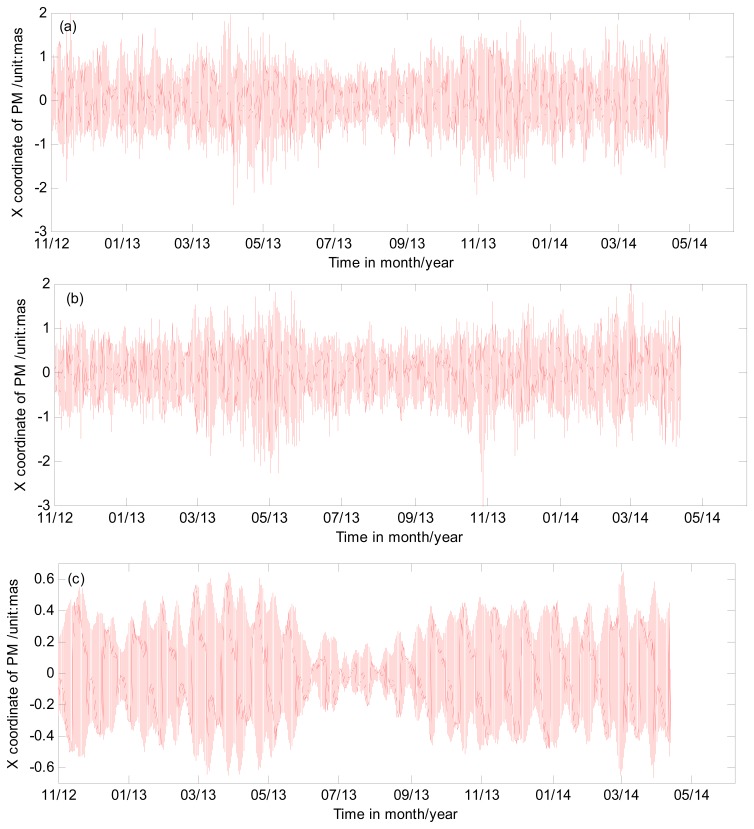
High frequency variations of ERP series generated from GPS with two hour sub-interval observations. (**a**) X-pole coordinate; (**b**) Y-pole coordinate; (**c**) UT1-UTC.

It is important to note that the series of high frequency variations of ERP contains both sub-daily variations and estimation error or other noises. The error from the empirical model we used and the detrending method may also alias into the high frequency variations. The high frequency variations in PM and UT1-UTC are shown in Figure 4 after removing the long period term. As seen in Figure 4, most of the high variations are within 1 mas in PM and 0.5 ms in UT1-UTC. Table 2 shows the detail statistics information of the high frequency variation series in PM and UT1-UTC. Furthermore, in order to analyze the impact of different sub-interval on the high frequency variations, ERP is also estimated with 4 h sub-interval observations. The statistic result of high frequency variations from four hour sub-interval are also given in Table 2.

As the results in Table 2 show, the combined GPS and GLONASS improves the precision of ERP when compared to GPS or GLONASS alone and the accuracy is improved again when ERPs are estimated using a long orbit arc of three days. In addition, the RMS of de-trend ERP is the minimum when ERP is generated from combined GPS and GLONASS with 3-day arc orbit by combining single day normal equations.

### 3.3. Analysis and Discussion

#### 3.3.1. Spectrum Analysis of Subdaily ERP from Different Satellite Systems

**Figure 5.**
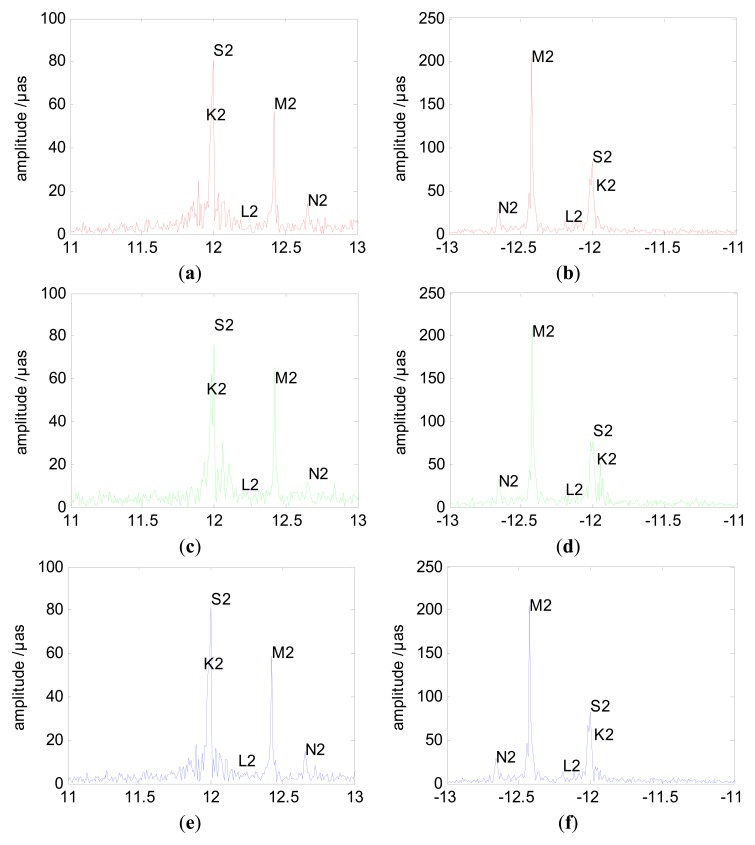
Amplitudes of prograde and retrograde semidiurnal PM from 2 h sub-interval. Prograde (**a**) and retrograde (**b**) semidiurnal PM from GPS, prograde (**c**) and retrograde (**d**) semidiurnal PM from GLONASS, prograde (**e**) and retrograde (**f**) semidiurnal PM from combined GPS/GLONASS.

To analyze the spectrum hidden in the series, the high frequency variations are converted to the frequency domain using a Fast Fourier Transform. Each high frequency variations series including X-pole, Y-pole and UT1-UTC can be expressed by a Fourier series independently as follows [17,24]:

(8) $$\begin{array}{l} \Delta Xp=\overset{k}{\underset{k=1}{\text{∑}}}[a_{k,xp}\sin\omega_{k}t+b_{k,xp}\cos\omega_{k}t]\\ \Delta Yp=\overset{k}{\underset{k=1}{\text{∑}}}[a_{k,yp}\sin\omega_{k}t+b_{k,yp}\cos\omega_{k}t]\\ \Delta (UT1-\text{UTC})=\overset{k}{\underset{k=1}{\text{∑}}}[a_{k,ut}\sin\omega_{k}t+b_{k,ut}\cos\omega_{k}t] \end{array}$$

where *ω_k_* is the frequency of Fourier series. The Nyqvist (maximal) and minimal frequency is 2*π*/2Δ*t* and 2*π*/*N*Δ*t*, respectively, where N is the smallest power of two that is greater or equal to the length of the series, Δ*t* is the temporal resolution of ERP series. Then the coefficients *a*_*i*,*xp*_, *b*_*i*,*xp*_, *a*_*i*,*yp*_, *b*_*i*,*yp*_, *a*_*i*,*ut*_ and *b*_*i*,*ut*_ of X-pole, Y-pole and UT1-UTC of Fourier analysis are converted to amplitudes as follows:

(9) $$A_{i,\text{amp}}=\sqrt{{a^{2}}_{i}+{a^{2}}_{i}}$$

(10) $$A_{i,\text{pro}}=\frac{1}{2}\sqrt{{\left(a_{i,xp}-b_{i,yp}\right)}^{2}+{\left(a_{i,yp}+b_{i,xp}\right)}^{2}}$$

(11) $$A_{i,\text{ret}}=\frac{1}{2}\sqrt{{\left(a_{i,xp}+b_{i,yp}\right)}^{2}+{\left(-a_{i,yp}+b_{i,xp}\right)}^{2}}$$

where *A*_*i*,*amp*_ is the amplitude of signal at the frequency *i*/*N*Δ*t* (*i* = 1,2,…N/2). *A*_*i*,*pro*_ and *A*_*i*,*ret*_ is the amplitudes of prograde and retrograde PM, respectively. First of all, we estimated the amplitudes of prograde and retrograde PM and UT1-UTC from different observations with 2 h sub-interval. As an example, Figure 5 shows the amplitude spectra of the PM at semidiurnal band from GPS, GLONASS and combined GPS/GLONASS. Several semidiurnal terms are marked in Figure 5. It is important to note that the terms in the figures are marked at proximate position. Since we only use a series of 526 days and no nodal factors are employed, the period of each terms listed here may be a little different form that of IERS Conventions 2010 [46]. Because the period of tidal terms are related to the frequency of Fourier series here. As Figure 5 shows, these sub-figures form GPS, GLONASS and GPS/GLONASS demonstrate very similar profiles. However, the exact size of amplitude of each terms is a bit different. Furthermore, the noise of GLONASS is a bit larger than that of GPS which also have a little impact on the size of amplitude. The amplitude spectral of UT1-UTC also shows the same result. But it is necessary to note that the amplitudes of these terms from UT1-UTC are extremely large when compared to IERS conventions. As we mentioned before, the reason is that we estimate UT1-UTC with only the first offset constrains to Bulletin A which leads to an imprecise ERPs. It also maybe a result of the presence of spurious signals. The coefficients of sine and cosine of PM and UT1-UTC from GPS, GLONASS and combined GPS/GLONASS are shown in the Table 3, respectively. Here, we just list 10 major terms. As the figures show, the coefficient from GPS, GLONASS and combined GPS/GLONASS are a bit different. The most difference occurs at the terms which are close to 24 h or 12 h. This will be discussed in detail based on the prograde and retrograde PM in the following when compared to result from IERS conventions and other researches.

To operate an appropriate comparison for these tidal terms, we firstly sum all the terms in the IERS tidal model and evaluate these whole tidal variations at the same epochs as our ERP series. The whole tidal variation series is regarded as a reference tidal variation series which then will be processed with the same method applied in our ERP series including the de-trending and FFT. Table 4 shows the amplitude of the prograde and retrograde PM at both diurnal and semidiurnal bands. In Table 4, we only list some main periodic terms, including both diurnal terms and semidiurnal terms. Furthermore, because the retrograde diurnal terms in PM are blocked in the IERS tidal model, these retrograde diurnal terms are not compared here. But for the semidiurnal terms, both prograde and retrograde are shown in the Table 4. As we can see, the largest difference for GPS and GLONASS occurs at 11.967 h (which corresponds to the semidiurnal term K2) and 24.058 h (which corresponds to the diurnal term P1) and 24.023 h (which corresponds to the diurnal term S1).

The differences of other terms whose period are not close to 12 h or 24 h are relatively small, but still exist. The most possible explanation is the different orbital period of GPS satellite (about 12 h) and the period of GLONASS (about 11 h 15 min). Although there is no algebraic relation between the orbital elements and tidal terms, the prograde tidal terms correspond to the translation of the orbital plane of each satellite. That is to say, the systematic changes of satellite in the orbit elements will lead to changes in the prograde tidal terms. For the GPS satellite, the period is about 12 h and the revolution is about 24 h in the inertial system, which is a systematic signal that propagates into those tidal terms with the same period. Furthermore, we can see that the terms of GNSS (combined GPS and GLONASS) at 24.058 h and 24.023 h is improved when compared to GPS only, because the difference between GPS and IERS is larger than that of GNSS. However, for those terms which are not so close to 12 h or 24 h (e.g., 26.859 h, 25.802 h), the combined result is dominated by GPS. In total, the standard deviation of the difference between GPS only and IERS 2010 is 18.33 μas. While the standard deviation of the difference between GNSS and IERS 2010 is 17.93 μas, which means the combined result is a little bit better than GPS only, overall. As a conclusion, the GPS satellite system has little impact on those tidal terms whose period is close to 24 h and 12 h. Thus, involving different satellite systems can reduce this impact in the theory, but our result is far to certify this adequately which need more investigation in the future.

#### 3.3.2. Spectrum Analysis of Subdaily ERP from Different Lengths of Sub-Intervals

**Figure 6.**
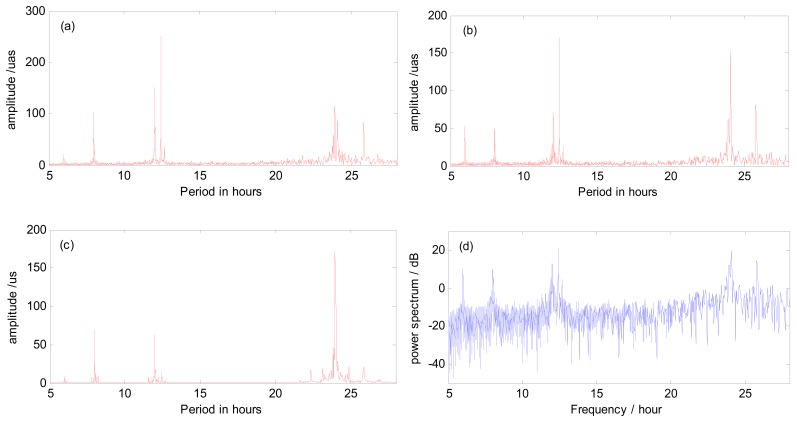
Spectrum analysis results of high frequency variations in ERP from combined GPS and GLONASS. Spectrum analysis of X-pole (**a**); Y-pole (**b**) and UT1-UTC (**c**); and power spectrum analysis of X-pole (**d**).

In this manuscript, we also estimated the ERP with 4 h sub-interval, namely ERP at a temporal resolution of 2 h. Figure 6a–c shows the amplitude spectrum analysis results of high frequency variations generated by combined GPS and GLONASS from 4 h sub-intervals with 3-day arc orbit. The amplitude spectrum analysis results almost show the same period terms in the X-pole and Y-pole. The amplitude spectral results of UT1-UTC show slightly different period terms than PM. Again, the amplitudes of these terms from UT1-UTC are extremely large when compared to IERS conventions. As we mentioned before, the reason is that the accuracy of UT1-UTC we estimated is not as good as PM due to the strategy we used. Other spurious signals may also be responsible for this. However, most of these periods are located around about 8 h, 12 h (semidiurnal variations) and 24 h (diurnal variations). Besides, we also get the detailed information about the amplitude of the noise as well as every period term. In addition, power spectrum analysis is also introduced to acquire a basic understanding of the energy distribution through all the period terms. As an example, the X-polar power spectrum analysis result is shown in Figure 6d. As the sub-figure shows, the power spectral peaks show almost the same result as the amplitude spectrum in that the main period terms are clustered together at one third day, semidiurnal and diurnal bands. As we may see, the power spectrum also shows a visible noise fluctuation. The most reasonable explanation is that when analyzing the power spectrum the whole series is regarded as a complete signal. If we divide the whole series into several pieces, the fluctuation can be smoothed.

**Figure 7.**
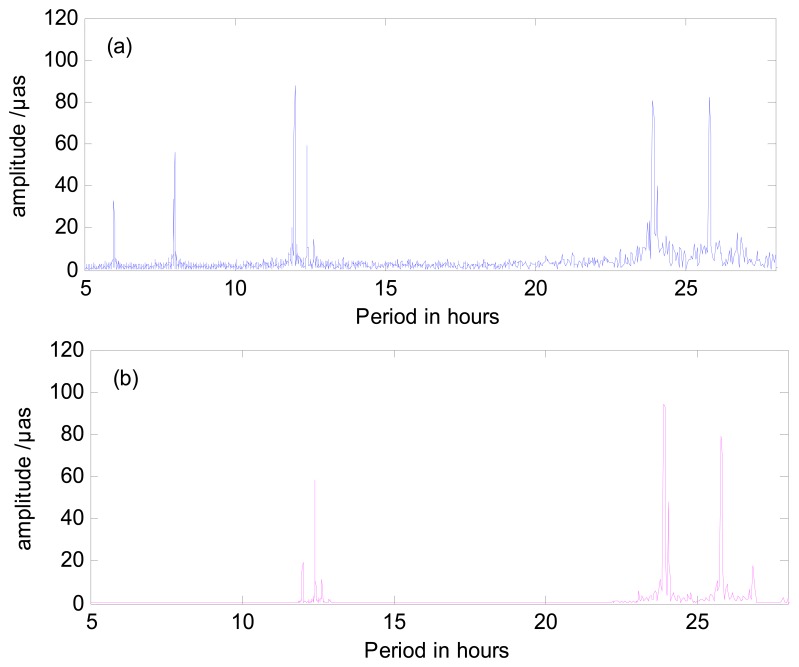
Amplitudes of prograde diurnal and semidiurnal PM. (**a**) prograde PM of our result from combined GPS/GLONASS with 4 h sub-interval, (**b**) prograde PM of from IERS convention 2010.

Furthermore, in order to analyze the impact of the length of sub-interval on the tidal amplitudes generated from the high frequency variations, the amplitudes of high frequency variations are estimated from both 2 h sub-interval and 4 h sub-interval. The amplitudes of prograde, retrograde PM and UT1-UTC generated from 2 h and 4 h sub-interval are compared. As we all know, the period terms (including diurnal and semidiurnal variations) generated from the high frequency variations are mainly the consequence of redistribution of geophysical fluid mass, especially the ocean tide. The tidal potential generated by moon and sun changes the ocean currents that cause the redistribution of earth fluid mass, which in return caused the variation in earth rotation. For instance, the period of 1.0024 days we obtain from the high frequency variations in ERP is one of the components of the ocean tides (which may correspond to *P*_1_ in IERS tide model). As a comparison, the prograde PM from IERS convention 2010 is shown in Figure 7 as well as our result. As Figure 7 shows, we obtain dozens of diurnal terms and semidiurnal terms. In addition, we also acquire several small terms, which are distributed around 1/3 d and 1/4 d. These short period terms are not included in the ocean tide model. Some of these short terms may be caused by the data processing method we use, but the most possible explanation is artifact. This has already been proved by analyzing the formal error spectra [23].

Table 5 shows the amplitudes of major period terms of PM and UT1-UTC obtained from high frequency variations. Most of these period terms listed below are included in ocean tide model in the IERS conventions 2010. In Table 5, we list 27 terms in total and the retrograde diurnal terms in PM are not shown here since they need to be blocked as we mentioned before.

Some of the period of these terms may be slightly different from the IERS Conventions. The most reasonable explanation is these period terms are not separated correctly due to the length of data we use. For example, the semidiurnal term of λ2 (0.5092406d) and L2 (0.5079842d) from the IERS might merge into one term at the period of 0.5079 d in our result. This should be improved if more than years of GNSS data were processed. For instance, to separate the largest 30 tidal terms completely given in IERS Convention, at least three or more years of GNSS data are needed. In the Table 5, the arrow (↑) (↓) means that the amplitude for the same tidal term computed from 2 h sub-interval is larger (smaller) than the amplitude computed from 4 h sub-interval. As the values listed in these tables show, 22 tidal amplitudes increase while 14 of them decrease in PM. It is also visible in UT1-UTC since 15 tidal amplitudes increase while 12 of them decrease. In conclusion, most of the amplitudes are increased when high frequency variations were generated from 2 h sub-interval GNSS observations. In another words, the amplitudes will be compressed or reduced when estimated ERP with a bit longer sub-interval. So in order to obtain the exactly amplitudes, it is better to determinate ERP at a higher frequency and shorter sub-interval as long as the precision is sufficient. However, the higher frequency is not necessarily better because it also relates to the accuracy of each parameter.

### 3.4. Influences of Different Models and Strategies

Apart from the time interval, there are various other factors that may impact on the estimation of ERP as well as the amplitudes of tidal terms. In this section, the influence of the prior information of ERP, ocean loading model, mapping function, and weighting function are discussed. Table 6 shows the impacts of different models or strategies on ERP. The factors we compared are (a) Bulletin A and C04 we use as prior information when estimating ERP; (b) ocean loading model (FES2004) and without ocean loading model; (c) weighting function (COSZ) and without weighting function (namely equally weighted); (d) mapping function of NIELL and HOPFIELD [47] when deal with the troposphere delays [48,49]. As Table 6 shows, the prior information of ERP has the minimal impact on ERP that can be ignored, while the impact of weighting function and ocean loading model demonstrate a visible impacts of different on ERP. The possible reason is that we set the cutoff angle at a low level (10°). When the elevation angle is small, the effect of multipath for some sites where there are many trees around (e.g., wuhn) becomes larger. Then the weighting function will have a visible impact on the estimation of parameters. So, as we suggested, weighting function and ocean loading model should be seriously considered when estimate ERP. As regarding to the mapping function, it also shows a very little influence.

The changes in ERP will leads to changes in the high frequency variations. Thus, the impact on amplitudes is also analyzed. Table 7 shows the impacts of difference processing options on tidal amplitudes. Here we only consider several major tides since the frequency resolution is not high enough to separate some ocean tides which are very close to each other. As we can see, Table 7 shows almost the same result as Table 6. Weighting function has shown a significant influence on the tidal amplitudes, while ocean load model also has a slight influence. As for prior information of ERP and mapping function, it shows a tiny difference. Other than these factors, the Reference Frame, sampling and orbit model may also have a slight impact on tidal amplitudes [23,28]. Here we did not cover all of them due to space reasons.

## 4. Conclusions

In this paper, the hourly ERPs time series are determined using GPS, GLONASS, and combining GPS and GLONASS data collected from 1 November 2012 to 10 April 2014. The observations come from nearly 80 IGS sites with a globally uniform distribution. We assess the precision of our ERP results by comparing them to the IGS solution. Our findings indicate that the accuracy of ERP can be improved by combining GPS and GLONASS. In addition, the correlation between the orbit model and ERP will also be reduced by combing these two different navigation systems. The high frequency variations in ERP are further studied using a de-trending method based on smoothness priors operating, like a time-varying FIR high pass filter. Our results show that most of the high frequency variations are within 1 mas for PM and within 0.5 ms for UT1-UTC. Furthermore, the spectrum of high frequency ERP variations is analyzed using a Fast Fourier Transform. As a result, we obtained several period terms for both PM and UT1-UTC around about 8 h, 12 h and 24 h bands. Most of these terms are components of ocean tides. By comparing the amplitudes generated from different time intervals, the result shows that the amplitude will be compressed or reduced when estimated ERP with a bit longer time interval. The amplitudes generated from different satellite systems have also been discussed. The results show that different orbits do have some effects on the tidal terms as we show with some figures as evidence, but our results are far from being able to certify this adequately and how this impact exactly happens need more investigation in the future. In addition, there are also some small terms that occurred in the high frequency variations of ERP. This may be caused by the models and strategies that we used in the data processing as well as artifacts. Some differences in the amplitudes from IERS may be caused by other geophysical excitations, such as the thermally driven atmospheric and high-frequency hydrological variations, which will be analyzed in the future with more geophysical data.

## Figures and Tables

**Table 1.** Difference between our daily averages of ERPs and IGS values.

	**PM in X/mas**			**PM in Y/mas**			**UT1-UTC/ms**		

	**Max**	**Min**	**STD**	**Max**	**Min**	**STD**	**Max**	**Min**	**STD**
GPS only	1.5465	−1.7256	0.4079	1.6519	−1.2389	0.4609	0.1071	−0.4033	0.0842
GLONASS only	2.2049	−1.8712	0.6763	2.4367	−2.3954	0.6834	0.0953	−0.5087	0.1021
GPS/GLONASS	1.5632	−1.7111	0.3832	1.4584	−1.1252	0.4301	0.1001	−0.4140	0.0833

**Table 2.** Statistics result of high frequency variations of ERP from different observations.

		**PM in X/mas**			**PM in Y/mas**			**UT1-UTC/ms**		

		**Max**	**Min**	**RMS**	**Max**	**Min**	**RMS**	**Max**	**Min**	**RMS**
2 h	GPS only	1.5293	−1.5849	0.4597	1.7599	−1.7781	0.4112	0.6151	−0.6398	0.2379
	GLONASS only	2.2588	−2.8308	0.5609	2.0416	−2.1159	0.5404	0.6120	−0.6541	0.2409
	GPS/GLONASS	1.5637	−1.4488	0.4423	1.4535	−1.4054	0.3891	0.5933	−0.6253	0.2348
	3 days arc	1.4471	−1.7828	0.4477	1.5848	−1.1956	0.3630	0.5893	−0.5846	0.2329
4 h	GPS only	1.7325	−1.763	0.4673	1.5932	−1.5914	0.3857	0.6031	−0.6091	0.2385
	GLONASS only	2.2294	−2.5976	0.5535	2.2496	−1.7075	0.5026	0.6031	−0.6685	0.2401
	GPS/GLONASS	1.4408	−1.3897	0.4504	1.3427	−1.2922	0.3680	0.5890	−0.5966	0.2340
	3 days arc	1.4012	−1.4215	0.4374	1.3648	−1.1626	0.3545	0.5788	−0.5921	0.2331

**Table 3.** Coefficients of sine and cosine of PM and UT1-UTC form different systems.

**Frequency of FFT/2*π*·*d*^−1^**	**Period/h**	**X-pole/μas**		**Y-pole/μas**		**UT1-UTC/μs**	

		**Cos**	**Sin**	**Cos**	**Sin**	**Cos**	**Sin**
Coefficient generated from GPS observations							

0.03723	26.859	1.467	−15.679	14.017	1.073	−3.50	1.69
0.03876	25.802	72.239	40.790	−11.676	79.050	4.86	−10.96
0.04156	24.058	−15.409	−100.467	−137.990	−40.446	73.33	−18.50
0.04163	24.023	−10.194	−32.503	14.023	22.829	110.24	34.56
0.04181	23.918	−21.341	−118.263	34.166	−60.994	−38.18	30.43
0.04187	23.883	12.936	2.107	−10.607	−0.289	4.38	4.84
0.07898	12.662	26.751	−28.465	−23.818	1.166	2.75	−1.08
0.08051	12.421	−21.771	247.721	160.688	−52.034	−5.65	14.07
0.08331	12.003	−115.643	98.433	52.203	−39.150	−69.59	17.48
0.08356	11.967	−17.791	−7.095	17.330	22.052	−8.64	−1.32

Coefficient generated from GLONASS observations							

0.03723	26.859	0.376	−10.878	8.841	−15.765	−3.66	1.58
0.03876	25.802	80.968	38.645	−6.116	75.489	5.05	−11.05
0.04156	24.058	−48.380	−89.783	−168.672	−20.558	70.73	−13.14
0.04163	24.023	6.600	4.006	−6.790	7.666	102.72	33.18
0.04181	23.918	−61.392	−105.817	28.908	−26.770	−37.08	26.60
0.04187	23.883	−15.947	23.678	−12.532	26.309	4.39	4.73
0.07898	12.662	26.652	−27.803	−29.961	−4.131	2.66	−1.28
0.08051	12.421	−33.054	252.585	171.502	−69.196	−5.21	14.34
0.08331	12.003	−121.566	57.062	67.587	−30.709	−46.14	11.70
0.08356	11.967	−19.862	−47.684	1.254	12.439	−8.23	−0.25

Coefficient generated from combined GPS/GLONASS observations							

0.03723	26.859	1.300	−14.456	11.975	−0.178	−3.46	1.61
0.03876	25.802	75.395	36.629	−13.648	80.423	4.95	−11.00
0.04156	24.058	−24.198	−100.922	−150.297	−40.290	72.34	−16.68
0.04163	24.023	−4.638	−19.448	13.318	17.320	107.23	33.86
0.04181	23.918	−31.986	−113.014	28.017	−50.420	−38.00	28.82
0.04187	23.883	5.503	10.046	−13.297	9.722	4.12	4.82
0.07898	12.662	27.813	−26.343	−27.294	1.034	2.65	−1.20
0.08051	12.421	−24.588	248.340	162.571	−56.315	−5.57	14.13
0.08331	12.003	−126.292	84.260	50.162	−32.255	−59.07	14.64
0.08356	11.967	−22.706	−19.336	14.093	15.941	−7.85	−1.33

**Table 4.** Amplitudes of prograde and retrograde PM from different models. The unit is μas.

**Frequency of FFT/2*π*·*d*^−1^**	**Period/h**	**Gps**	**Glonass**	**Gnss**	**Iers 2010**

		**Prograde**	**Prograde**	**Prograde**	**Prograde**

		**Retrograde**	**Retrograde**	**Retrograde**	**Retrograde**
0.03723	26.859	14.902	12.507	13.228	17.975
0.03876	25.802	80.064	81.367	81.865	79.081
0.04156	24.058	33.644	52.383	40.610	48.038
0.04163	24.023	24.105	8.945	17.568	3.416
0.04181	23.918	86.622	80.504	81.671	94.152
0.04187	23.883	8.967	18.832	13.935	15.268

0.07898	12.662	14.150	11.312	14.5431	11.388
		29.104	32.727	29.975	29.180

0.08051	12.421	57.057	65.249	58.952	58.288
		204.765	212.812	206.067	206.179

0.08331	12.003	80.775	76.319	81.068	19.649
		84.473	77.124	82.025	89.557

0.08356	11.967	12.397	24.749	17.053	4.382
		20.568	28.280	19.501	20.549

**Table 5.** Tidal Amplitudes of PM. ↑ represents increased, ↓ represents decreased.

**Frequency of FFT/2*π*·*d*^−1^**	**Period/days**	**Amplitude from 4 h Sub-Interval**			**Amplitude from 2 h Sub-Interval**		

		**Prograde PM/μas**	**Retrograde PM/μas**	**UT1-UTC μs**	**Prograde PM/μas**	**Retrograde PM/μas**	**UT1-UTC μs**

Diurnal terms in PM										

0.03723	1.1191	15.60		4.53	15.88 ↑		4.00 ↓
0.03741	1.1136	16.47		2.25	18.79 ↑		2.30 ↑
0.03876	1.0751	81.33		10.44	80.08 ↓		10.55 ↑
0.04028	1.0343	14.61		7.85	13.49 ↓		7.52 ↓
0.04053	1.0281	7.76		10.46	7.83 ↑		10.64 ↑
0.04071	1.0235	5.52		2.78	3.44 ↓		3.30 ↑
0.04144	1.0054	29.28		17.09	30.55 ↑		17.70 ↑
0.04150	1.0039	18.27		24.23	17.68 ↓		23.56 ↓
0.04156	1.0024	45.89		75.46	50.18 ↑		75.57 ↑
0.04163	1.0010	24.98		115.94	26.41 ↑		115.59 ↓
0.04169	0.9995	13.50		181.02	18.81 ↑		181.14 ↑
0.04175	0.9981	73.76		35.97	73.85 ↑		36.20 ↑
0.04181	0.9966	81.77		53.97	80.60 ↓		53.40 ↓
0.04187	0.9951	18.55		6.53	20.36 ↑		6.87 ↑
0.04205	0.9908	13.70		8.56	13.83 ↑		8.09 ↓
0.04303	0.9683	4.69		6.61	5.69 ↑		6.18 ↓
0.04474	0.9313	7.04		18.02	7.54 ↑		18.47 ↑
0.04633	0.8994	2.73		1.70	3.22 ↑		1.15 ↓

Semidiurnal terms in PM										

0.07898	0.5276	13.93	28.41	3.17	13.73 ↓	29.61 ↑	3.09 ↓
0.08051	0.5176	60.15	204.04	15.87	60.67 ↑	203.24 ↓	15.95 ↑
0.08203	0.5079	6.79	10.06	3.89	7.42 ↑	9.70 ↓	3.64 ↓
0.08319	0.5009	18.88	72.00	15.33	21.27 ↑	71.42 ↓	15.27 ↓
0.08325	0.5005	10.63	52.81	10.59	10.77 ↑	51.56 ↓	10.88 ↑
0.08331	0.5001	77.81	89.58	61.38	83.89 ↑	88.47 ↓	61.77 ↑
0.08344	0.4994	43.52	24.42	26.38	46.13 ↑	21.57 ↓	26.50 ↑
0.08350	0.4990	41.44	3.21	8.51	41.12 ↓	3.54 ↑	8.18 ↓
0.08356	0.4987	16.48	21.79	8.13	16.41 ↓	24.04 ↑	8.43 ↑

**Table 6.** Impact of different GNSS models on ERP. (**a**) Bulletin A and C04; (**b**) ocean loading model and without ocean loading model; (**c**) weighting function and without weighting function; (**d**) mapping function of NIELL and HOPFIELD.

**GNSS Models**	**Difference in X-Pole/mas**		**Difference in Y-Pole/mas**		**Difference in UT1-UTC/ms**	

	**Largest**	**RMS**	**Largest**	**RMS**	**Largest**	**RMS**
(a)	0.240	0.062	0.030	0.096	0.052	0.018
(b)	0.470	0.173	0.830	0.280	0.040	0.016
(c)	1.360	0.655	2.120	0.842	0.367	0.114
(d)	0.230	0.070	1.090	0.201	0.020	0.007

**Table 7.** Impact of different GNSS models on tidal amplitudes. (**a**) Bulletin A and C04; (**b**) ocean loading model and without ocean loading model; (**c**) weighting function and without weighting function; (**d**) mapping function of NIELL and HOPFIELD.

**GNSS Models**	**Amplitudes in X-Pole/μas**		**Amplitudes in Y-Pole/μas**		**Amplitudes in UT1-UTC/μs**	

	**Largest Difference**	**RMS**	**Largest Difference**	**RMS**	**Largest Difference**	**RMS**
(a)	3.17	1.73	7.41	4.02	0.45	0.27
(b)	9.14	4.56	7.81	5.94	0.92	0.55
(c)	19.93	10.21	59.63	33.28	13.83	8.77
(d)	4.33	2.07	13.22	6.02	0.76	0.43
